# New Media, Digitalization, and the Evolution of the Professional Sport Industry

**DOI:** 10.3389/fspor.2022.921329

**Published:** 2022-06-13

**Authors:** Jingxuan Zheng, Daniel S. Mason

**Affiliations:** Faculty of Kinesiology, Sport, and Recreation, University of Alberta, Edmonton, AB, Canada

**Keywords:** information economy, attention economy, multi-sided market, value co-creation, ecosystem

## Abstract

The professional sport industry achieved tremendous success in the traditional broadcast media age, established a multi-sided market and an effective business model for revenue growth. However, the emergence and proliferation of the new media technologies have drastically changed the media landscape, creating a much more complicated cross-media environment that unites popularity and personalization, structure and agency. Such a changing environment creates transformations within the professional sport industry, and adapting to these transformations will lead to the evolution of the professional sport industry and its success in the digital media age.

## Introduction

The professional sport industry has thrived with the formation of a symbiotic relationship with traditional mass media (Gratton and Solberg, 2007) and a derived multi-sided market model, characterized by a synergistic effect of value creation from both the demand and supply side. However, the advent and proliferation of new media technologies have profoundly changed the media landscape and its compatibility with the professional sport industry and disrupted the value creating effectiveness of the mass-media-centric sport multi-sided market. This conceptual paper examines how digitalization and new media transformed the multi-sided market of professional sport industry. To do so, we develop an alternative cross-media ecosystem model of sport based on recent communication studies and uncover four major implications that the sport industry might face due to the transformation of its external environment into a cross-media ecosystem characterized by the synergistic effect of traditional mass media and myriad emerging digital technologies.

This paper contributes to the literature on digitalization occurring within a sport management context. Despite the rapid advancement of digital technology in broader society and its prevalent applications in the sport context, sport management research regarding digital transformation of the sport industry is still in its infancy. Currently the main issue facing digital transformation in sport management research relates to the lack of a theoretical framework to unify studies pertaining to traditional mass media and new media, as well as the new ecosystem emerged from changing relationships between sport business and their divergent types of media partners. In this paper, by developing a theoretical framework of a cross-media ecosystem, we offer a useful tool to systematically analyze the impact of digital transformation on the sport industry and unveil new value creating mechanisms that can lead to continuous prosperity of professional sport industry in the new media age.

The remainder of this paper is structured as follows. With a focus on the North American context, we first review the relationships between the professional sport industry and the traditional mass media, to explain the value creating mechanism of the sport multi-sided market. We then examine the disruptive changes that had happened in the sport-media symbiotic relationship due to the rapid growth of new media technologies, and developed an alternative cross media ecosystem model to analyze the evolutionary implications of digital transformation for professional sport industry. We conclude by identifying future avenues for digital transformation in sport research.

## Professional Sport and Traditional Mass Media: A Value Creating Multi-Sided Market

The professional sport industry formed early ties to the mass media when newspapers and other print media started to report game-related information to readers (Walker, 2015). Since the 1950s, with the advent of television, the professional sport industry has seen enormous revenue growth outside of traditional gate revenues (Wenner, 1989). The synergies between the media and the professional sport industry created a lucrative business model for both industries, boosting their potential to reach and establish new audiences. In this section, we introduce the multi-sided media-sport business model and analyze how the alliance between sport and media has benefitted both.

### Mass Media and the Formation of a Multi-Sided Market

With its emergence in the late nineteenth and early twentieth centuries, professional sport initially depended on gate receipts as the primary source of income (Fort, 2018). However, with the development of new mass media technologies (particularly television), media rights income has become the single most important revenue for the professional sport industry (Gratton and Solberg, 2007; Zheng and Mason, 2018). Multi-sided markets refer to “markets in which one or several platforms enable interactions between end-users and try to get the multiple sides ‘on board' by appropriately charging each side” (Rochet and Tirole, 2006, p. 645). Multi-sided market owners face a classic “chicken and egg problem,” which forces them to take on the strategy of subsidizing one side of a market in order to draw revenues from the other side to the market, ultimately resulting in the market's financial viability (Wright, 2004). For example, as a typical two-sided platform, eBay charges online vendors listing fees and/or commissions on one side of the market, while granting free access for consumers on the other. As more consumers enter the platform, vendors become more interested in paying to reach customers through the platform; in turn, the more vendors (and purchase options) consumers can access, the greater the number of consumers will use eBay for online shopping. This mechanism is defined as the network effect or, to be more specific, the *cross-side network effect*, which occurs where the more users exist on one side of the market, the more utility users on the other side of the market obtain from joining the network (Eisenmann et al., 2011). Meanwhile, *same-side network effects* enable users to reap more benefits by simply accessing a larger network of users (Economides and Tåg, 2012), such as with the telephone or internet.

**Figure 1:**
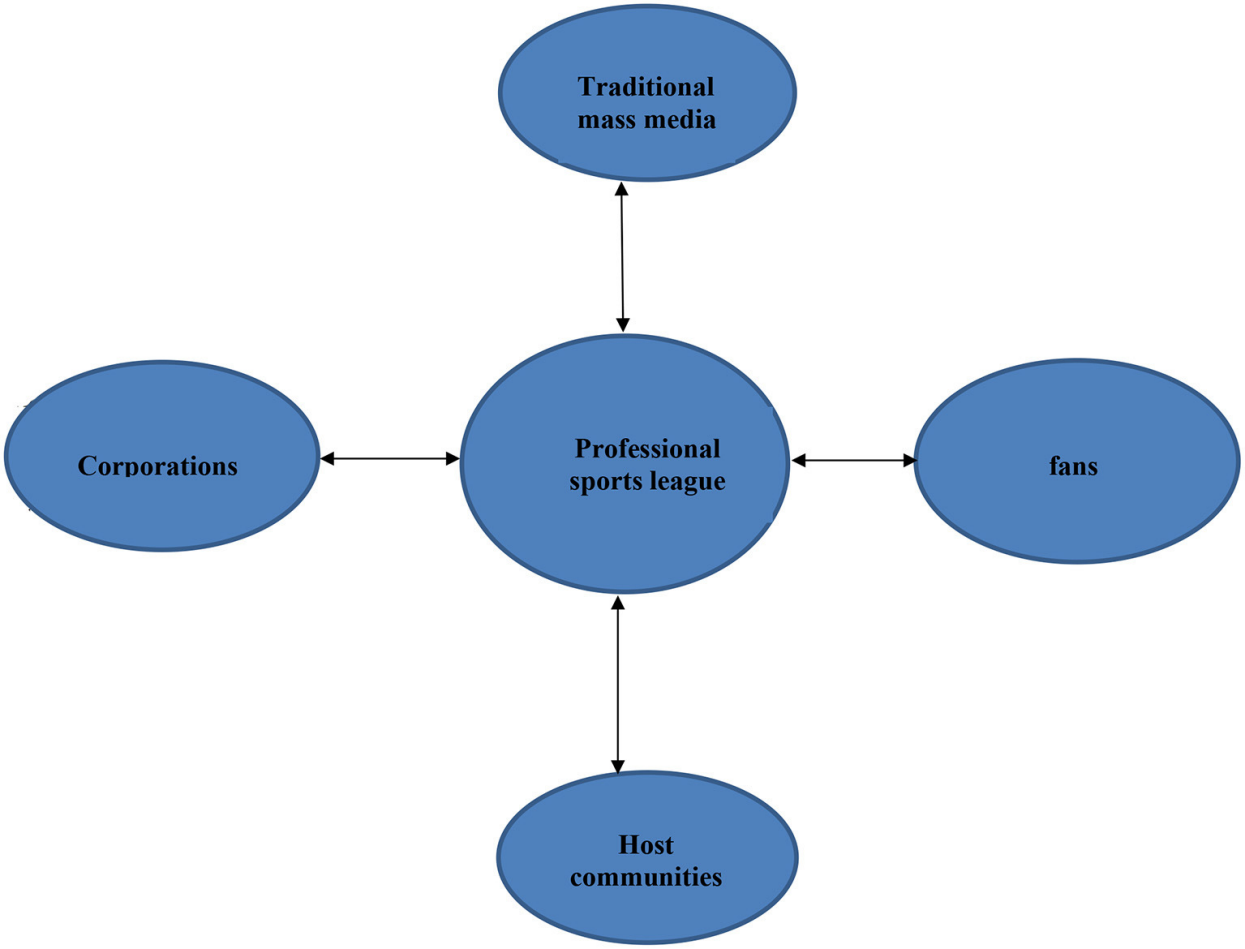
Basic model of professional sport multi-sided market with traditional mass media (adapted from Benner and Tushman, 2003).

Professional sport has developed into a special type of multi-sided market (see Figure 1) consisting of the league as the central platform and the fans, the media, corporate sponsors and host communities on different sides (Mason, 1999; Zheng and Mason, 2018). Leagues generate revenues from: fans primarily through gate revenues; the media *via* broadcast rights; corporate sponsors with sponsorship fees; and host communities *via* subsidies provided for facilities (Mason, 1999). However, professional sport is a special type of multi-sided market in two ways. First, instead of a conundrum between the “chicken” and the “egg,” sport fans—more specifically, gate-paying spectators—were the side that generated the initial momentum for professional sport to develop into a multi-sided market. As sport's fan base grew, the media, corporate sponsors, and host communities were drawn to the platform and provided different sides of it (see Figure 1).

Second, across the network of the professional sport multi-sided market, the media magnify the network effects with and across the different sides. For instance, more media coverage will attract more corporate sponsors and intensify the competition between host communities competing for the right to host sport teams.^1^ With the magnifying effects of the mass media, the professional sport industry was able to fully exploit the network effects generated from the different sides of the market, and turned these network effects into a “double whammy” (Shapiro and Varian, 1998, p. 182)—the combination of both demand-side economies (economies of networks) and supply-side economies (economies of scale).

### Information Product and the “Double Whammy”

Mason (1999) argued that the core product of professional sport industry is the uncertainty of the game outcome, or the game itself. However, with the formation of a symbiotic relationship between the profession sport industry and the mass media, this core product has become mediated, where a significant portion of the product is consumed as content available through the media. Once produced, the mediated sport product can be replicated, edited, repackaged, and distributed according to consumer tastes for very little incremental cost.

On the demand side, same-side network effects among fans can be significant, where the more fans watching the game, the more valuable the game because the same game experience can be shared with more people (Zheng and Mason, 2018). Moreover, unlike other products that have diminishing marginal utility, the professional sport product has an increasing marginal utility due to the fact that the more a fan follows a certain sport, the more knowledge she/he will possess regarding that particular sport, and the more she/he will be able to enjoy the experience (Dietl et al., 2012). These strong same-side network effects generated among fans are magnified by the mass media, and cross different sides of the professional sport multi-sided market, creating strong cross-side network effects as well among the media, corporate sponsors and host communities (Zheng and Mason, 2018). Combined, the same-side network effects and the cross-side network effects engender a *positive feedback loop* (Shapiro and Varian, 1998), expand the professional sport multi-sided market even more, and in the end, concentrate the market to a winner-take-all (Cook and Frank, 2010) structure. This process is what has allowed for some powerful media providers and sports leagues to emerge that have dominated the industry.

### The Domination of Sport-Media Conglomerates

With network effects engendered by the mass media on the demand side, and the mediated sport products produced by the mass media on the supply side, a combination of supply side economies of scale and demand side economies of network created a professional sport market dominated by a select few (Zheng and Mason, 2018). This can be attributed to the centralized, one-to-many nature of the traditional mass media (Napoli, 2010), which came to be dominated by a few television networks. Through a top-down broadcasting mode that distributed homogeneous content, a few media providers “enjoy[ed] exclusive formal and informal access to elite sources and act as gatekeepers by filtering information that they consider newsworthy and disseminating it to the general public” (Etter et al., 2019, p. 31). The value of sport content was rooted in *broadcast scarcity* (Hutchins and Rowe, 2009), where viewers had few options to choose from and sport was a form of media content that could attract significant demand.

## Emerging Digital Media: A Cross Media Ecosystem Model

In contrast to a few traditional mass media outlets producing and disseminating homogeneous content through a top-down, one-to-many process, with a stark structural distinction between the gatekeeping role played by the mass media and a passive information receiver role played by the audiences (Etter et al., 2019), new media—more precisely, social media—fundamentally changed the media domain. In this section, we identify and analyze those changes that have had an impact on the way information is disseminated and how society functions as a whole. First, however, we need to distinguish between new media and traditional mass media, and differentiate types of new media.

### Typology of New Media

Not all of the types of new media that emerged were completely different from, or more advanced than, the traditional mass media. For instance, Web 1.0 new media refers to the nascent generation of Internet websites that has a similar information distribution mode as the traditional mass media, which is characterized by a one-to-many broadcasting distribution structure (Drury, 2008). Web 1.0 new media “might be likely to adopt many of the characteristics of traditional mass media rather than evolve as the entirely unique and revolutionary medium” (Napoli, 2010, p. 56). In this instance content production and platform formation have usually been singlehandedly controlled and distributed by one organization, with little user interactivity (Filo et al., 2015).

In contrast, Web 2.0 new media, or social media, can be defined as “a group of internet-based applications that build on the ideological and technological foundations of Web 2.0, which facilitates interactivity and co-creation that allow for the development and sharing of user generated content among and between organizations and individuals” (Kaplan and Haenlein, 2010, p. 61). Facebook, YouTube, Twitter, Instagram and Snap are examples of Web 2.0 new media, or social media. Social media is characterized by heterogeneous, user-generated-content (UGC), horizontal information co-creation and distribution, and selective exposure and audience fragmentation (Etter et al., 2019). Social media users “engage in participatory and collaborative content generation by sharing, linking, collaborating, and producing online content using text, photo, audio, and video” (Abeza et al., 2015, p. 602). It is New Media 2.0, or social media, that has the greatest potential impact on the traditional multi-sided market structure of professional sport.

Recent years have witnessed the emergence and development of web 3.0, which remains in its nascent stages; hence the denotation and connotation of what is web 3.0 is still heatedly debated among practitioners and scholars. However, two widely recognized and acknowledged characteristics of web 3.0 include: first, web 3.0 is constructed based on blockchain technology and the broad use of cryptocurrency, which enable decentralized interactions among internet users, rebuking centralized control of services and information from internet giants such as Facebook or Google in Web 2.0 (Kshetri, 2022); second, web 3.0 is characterized by an immersive, networked “metaverse” which can be generally described as a parallel virtual world built alongside the real world (Metaverse, 2022). With the advanced technology such as Artificial Intelligence (AI), Virtual Reality (VR), Augmented Reality (AR), or even holograms, people “live” in the “metaverse” vicariously through their avatars, and can engage in all kinds of activities such as virtual shopping, gaming, tourism, as well as sports entertainment and training (Gursoy et al., 2022; Metaverse, 2022). For instance, “the Manchester City club has become the first soccer club to announce that the Etihad Stadium will be virtually recreated in the Metaverse,” which “will allow fans of the soccer team to watch matches live without having to physically enter the stadium” (Metaverse, 2022, para. 20).

### Supply Side Change

This is because new media, especially social media, alters the way information is produced and distributed. Traditional mass media operates in a top-down mode to broadcast information from a few content providers to an audience of many (Petko et al., 2015) with limited opportunities for audiences to respond; hence it is easy to control content production and distribution channels, as well as the attributes of the content itself. Social media bring alternative ways to this vertical broadcasting model of the traditional mass media by enabling the emergence of a “bottom-up” model characterized by organized co-production, information creation, and dissemination (Etter and Nielsen, 2015). “Social media now enable vast audiences to serve as both senders and receivers” of information and to “collectively engage in the coproduction” of this information (Etter et al., 2019, p. 36). With social media, content is no longer produced by a few media providers, but co-created by actors from different backgrounds and viewpoints (Prahalad and Ramaswamy, 2004). Collectively, millions (billions) of users of Facebook, Twitter, Instagram, YouTube, and other social media comment on and alter the narratives and discourse of the information produced by the mass media, post their own related content, and expose or reveal events that may be neglected by the mass media, therefore subverting mass media's role as the gatekeeper of information and social rhetoric (Shoemaker and Vos, 2009). In the social media context, any individual that produces and diffuses information has the potential to gain widespread attention (Webster, 2016).

The emergence of social media has blurred old distinctions between information producers and senders, and information receivers (Etter et al., 2019), and enables the establishment of a horizontal “hyperlinked society” (Maeyer et al., 2013) where content is created in chunks of interconnected networks of the online world (Ellison and Boyd, 2013) and distributed in a nonlinear mode (Manovich, 2002). Every user of this networked online world has the potential to “contribute to the creation and rapid diffusion of content,” as they “freely and easily share information across and between different platforms” (Etter et al., 2019 p. 36). Even traditional mass media “increasingly rely on social media users as sources, using information circulated through social media channels for their reporting, which is then picked up by social media users again” (Etter et al., 2019, p. 36).

With the traditional mass media, homogeneous information was produced and diffused by a few media gatekeepers; in the social media age, content stems from different sources created by individuals or organizations with diverse motives (Etter et al., 2019). Users of social media produce content based on their own personal identities and experiences instead of abiding by the commercial news criteria of the traditional mass media, and this “experiential credibility” (Hussain et al., 2017) can be deemed more authentic and trustworthy than information promoted by giant media conglomerates reflecting “corporate interests” (Johnson and Kaye, 2004, p. 625). Social media users also have disparate motives to engage in information creation and dissemination. Individuals and organizations “use social media to build or reinforce a distinctive image—frequently built in opposition to corporate practices—by supporting or stigmatizing actions that are congruent or incongruent with the social values they advocate” (Etter et al., 2019, p. 38).

Additionally, the traditional mass media produce more objective information due to their necessity to conform to established industry norms and ethical codes (Deephouse, 2000), while social media content is often created to express strong emotional sentiments, such as “anger and frustration, surprise and excitement, shock and disgust, or joy” (Etter et al., 2019, pp. 39-40). Without the filtering process imposed by the traditional mass media, emotion-laden content prevails more readily in the online world, attracts rapid and wider public attention, and enables “emotional contagion” (Hatfield et al., 1993) to more like-minded individuals. With social media, “emotionally charged information shared by an original sender with their links rapidly branches out in multiple directions and indirectly reaches and possibly mobilizes a vast audience” (Etter et al., 2019, p. 40).

### Demand Side Change

In the new media age, homogenous information is gradually replaced by highly heterogeneous and idiosyncratic content created by diverse groups of actors. These actors selectively expose themselves to similar information “that confirms prior beliefs and to ignore disconfirming information” (Etter et al., 2019, p. 41) due to their autonomy in choosing information. Some recently developed new media technologies such as feeding algorithms reinforce this positive feedback loop by optimizing the chances of the audience being exposed to content with similar traits over and over again; meanwhile, even traditional mass media “offer their audiences increasingly narrow, partial, and preselected information” in order to “compete for the attention of niche audiences” (Etter et al., 2019, p. 41). The combination of effects of both individual's preferences for consistent information that can resonate with their existing beliefs and values and new media's tendency to cater to these preferences has led to a continuous fragmentation of the audiences and the formation of *reputation silos* (Pariser, 2012; Turow, 2012) and *echo chambers* (Sunstein, 2018). These echo chambers are “online spaces, such as fan forums or online activist communities, that host exchanges among like-minded individuals who are sheltered from opposing views” (Etter et al., 2019, p. 41).

Because of the existence of echo chambers and reputation silos engendered by the advent and proliferation of the new media technology, some communication scholars worry that society is becoming increasingly polarized and fragmented, where people screen out any dissonant information that runs the possibility of contravening their prior beliefs and values (Stroud, 2011; Levendusky, 2013). This will lead to the “growth of a massively parallel culture composed of millions of microcultures and tribal eddies” (Anderson, 2008, p. 183). However, Webster (2016, 2017) argued that people still tend to be drawn to the most popular informational content offered by whatever media platform is available, and built a model of exposure and a cross media ecosystem which integrates traditional mass media and new media.

### A Dynamic Model of Exposure and a Cross Media Ecosystem

**Figure 2:**
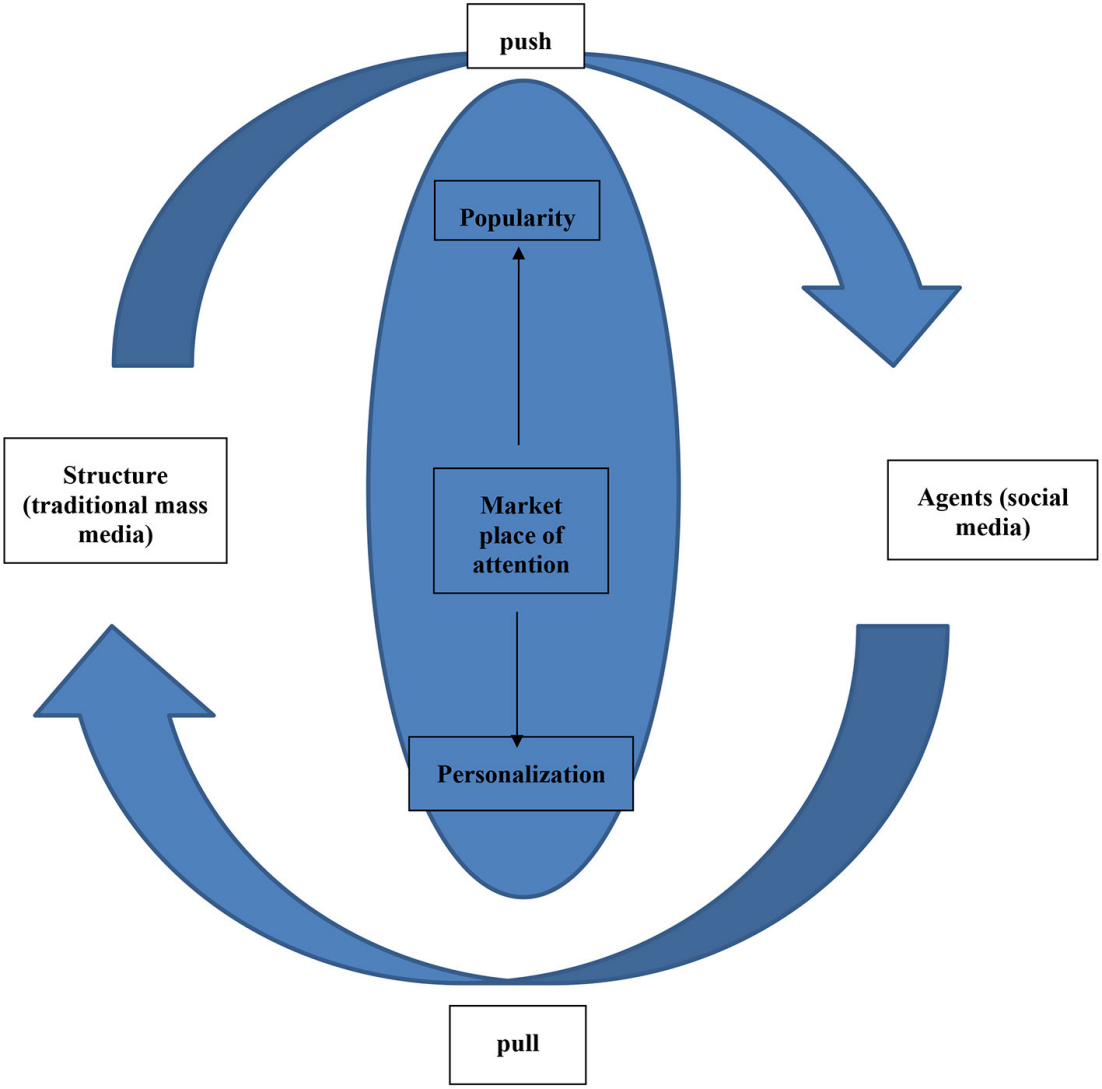
Model of a cross media ecosystem (adapted from Webster, 2017).

Media users now face a cross-media environment where traditional mass media and new media (social media) play their respective roles to meet the collective and individual needs of different users. In the dynamic model of exposure that represents such a cross-media market (see Figure 2), Webster (2017) suggested that through “user information regimes” (Webster, 2011) such as online recommendation systems, new media users *pull* certain types of information content toward them according to factors exogenous to the media context, such as their predisposed tastes, needs, attitudes, or moods; while *via* market information regimes (Anand and Peterson, 2000) like the Nielsen ratings services, and traditional mass media *push* particular kinds of contents toward the same media users whose preferences are determined by factors endogenous to the media context. In the new media age, users have unprecedented agency to pull similar patterns of content to them over and over again; however, new media users are embedded in a nonlinear, recursive media environment where media exposure is not only determined by personal preferences, but also influenced by structural factors such as big TV networks, famous journalists, advertisers, and online algorithms.

By and large, the new media age might be more precisely described with a cross media model (see Figure 2) where unification of the traditional mass media turns into a dichotomy of personality and structure, which both attempt to draw massive attention toward their information product, hence the establishment of “the marketplace of attention” (Webster, 2016).

## Implications For the Professional Sport Industry in a Cross-Media Ecosystem

In this section, we analyze the transformational implications of a cross-media ecosystem for professional sport industry. First, fans' consumption behavior has been altered by new media technology, which enables them to consume the holistic professional sport experience as opposed to discrete, time-restricted games; second, beyond consuming the content produced and distributed by the professional sport leagues and teams, new media users possess the tools to create their own forms of mediated content based on their own idiosyncratic demands, as well as express themselves more freely and immediately. Third, radical technological innovation of digitalization erodes the old business model of professional sports, highlights the strategic paradox between exploitation and exploration, and calls for the simultaneous exploitation of the broadcasting business model of a multi-sided market and exploration of a new media business model to deliver value to the customers, and to create new revenue flows for the sport industry. Finally, new media—especially social media—transformed how sport leagues or teams are viewed and discussed, creating unexpected turmoil where disruptive events have occurred. Next, we will break down these transformations in greater detail.

### Attention Economy and Changes to Viewing Behavior

**Figure 3:**
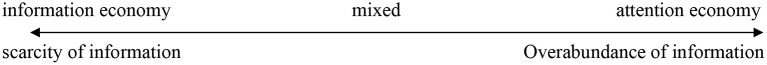
Information economy/attention economy continuum.

The attention economy perspective suggests that as new media technology has enabled abundant information to be produced on a daily basis, the term *information economy* is replaced by *attention economy*, where information is abundant and the scarce resource is the attention required to consume said information (Simon, 1991; Davenport and Beck, 2001; Lanham, 2007). With this logic, the attention economy and the information economy can be conceived as ends of a continuum (see Figure 3). As shown in Figure 3, the left side of the “information economy/attention economy continuum” represents a hypothetical information economy where information production is virtually nonexistent, and the collective human attentive capacity to consume that information is far greater than the information that is produced. As more information is produced—due to technological advancement, for example—it will eventually overwhelm the collective human attentive capacity to consume it at a certain point. Thus, society shifts to an attention economy. Real-life scenarios fall in between the two hypothetical cases, representing a mixed information and attention economy, and the dominance of either changes with the evolutionary course taken by technology. For instance, traditional mass media and sport exist in an information economy. With the overabundance of information being produced with the assistance of the new media technologies, traditional mass media started to integrate the attention economy by establishing and incorporating myriad new media platforms, which was combined with new sport-related content produced by other actors.

New media 2.0, or social media, exists in a predominantly attention economy, which has a profound impact on consumer behavior and demands the emergence of innovative business models to bring ongoing value to the sport fans who are new media users as well. Evidence has shown that entering the new media age, media users' attention span has narrowed (Davenport and Beck, 2001; Fidler, 2018) due to the attention deficiency when facing information overload. This has brought fluctuations in viewer numbers even for major professional sport leagues such as the National Football League (NFL). For instance, a 9.7% drop was reported in overall NFL ratings for the 2017-18 season, an even steeper decline from the 8% drop from the 2015-16 season (R/GA, 2018). Richard Ting, Global Chief Design Officer, R/GA, pointed out that “Nowadays, consumers have such fragmented attention spans. They have such limited time to devote to a 2-h-long basketball game or 3-h-long baseball game. Sports are competing with so many different things, like video games and YouTube videos.” (R/GA, 2018, para. 5).

In the new media age, sport fans are more likely to watch the game from multiple media platforms (Zheng and Mason, 2018); they are more likely to watch highlights than the entire game (R/GA, 2018); and they are more likely to watch sports games from non-linear media such as online streaming than linear ones such as television (Singer, 2017). For example, “more than 1.3 million homes in the US dropped traditional cable or satellite TV service in the second quarter of 2020” and switched sport viewership to over-the-top (OTT) delivery platforms such as DAZN, Amazon Prime Video, or OTT delivery service provided by traditional sports broadcasters such as Fox Sports or ESPN (How Sports, 2020, para. 1; Newman, 2020). Compared with paid cable and satellite TV services, these OTT platforms not only cost less for the consumers, but can also transcend the boundaries of broadcast media and allow sport viewers to “watch more sport on more devices in more places than ever before and to personalize viewing practices and experiences across a variety of screens and communities of interest” (Hutchins and Rowe, 2019, p. 977). In addition, fans also use social media platforms such as Facebook or Twitter to comment on the game or communicate with their friends during game time, while logging onto YouTube to watch game recaps and highlights beyond the game time. Kavanagh (2019) pointed out that in May 2019, “28% of live social video viewers—those who have used the live feature on Facebook, Instagram, Snapchat or Twitter—have commented about a sports event on Facebook and 17% have done so on Twitter” (Para. 21). In addition, “Globally, 22% of internet users say that following sports events is one of their primary reasons for using social networks, climbing to 39% among live social video viewers” (Kavanagh, 2019, para. 14).

New media technology-savvy fans also prefer to watch shorter content such as highlights or game recaps over entire games. Tom Richardson, founder and president of Convergence Sports & Media, described this *highlight economy*: “The league is putting out real-time highlights in-game, if you really want to pay attention as a fan, you can do that in a highlights environment, without watching the actual product” (R/GA, 2018, para. 8). Evidence also showed that the slipping viewership numbers for major sport leagues such as NFL or MLB might be attributed to the fact that fans are watching in fewer and shorter increments due to their dispersed attention toward so many different information sources (Singer, 2017). For example, the 9 percent ratings drop of the NFL in the 2016–17 regular season among millennials “was caused by an 8 percent drop in the number of games watched and a 6 percent decline in the minutes watched per game (down to 1 h 12 mins per game)” (Singer, 2017, para. 4). Confronting the rise of the new media technologies and the changing viewing behaviors of a younger generation of sport fans, professional sport leagues and traditional sport media strived to adapt to a more new-media-centric business model. For instance, “Major League Soccer replaced local cable rights with digital only local rights on platforms such as YouTube TV and ESPN+,” while “ESPN launched a streaming service that will replace live sport as the foundation of the cable industry” (Agha and Dixon, 2021, p. 24).

Webster (2016) suggested that a linear way of delivering content is characterized by a predetermined broadcast schedule by which audiences must abide. “Even with hundreds of channels delivered by cable and satellite, users may have to accommodate themselves to the schedule of linear delivery” (Webster, 2016, p. 64); whereas a nonlinear delivery system “let users fetch what they want at almost any time” (Webster, 2016, p. 64). Watching professional sport games on television is a typical linear way of consuming mediated sport product. With or without broadcasting, professional sport games only take place at a certain time, and because the game itself is time sensitive and perishable, watching a game after it ends considerably reduces its utility to consumers. Therefore, research shows that new media users, even millennials, still enjoy watching live sport games; and “more believe they have increased the amount of live sports they watch on TV than those who think they have decreased” (Singer, 2017, para. 10). However, we want to argue here that new media users are more likely to utilizie nonlinear media platforms to build themselves the whole sport experience; hence to a certain extent, the importance of the game itself and its uncertain outcome declines as an experiential product can still be devised and delivered without watching the entire game.

### Value Co-creation and Power Balance Tipping

In marketing research, a service dominant logic (SDL) suggests that consumers create and determine their own value-in-use with the value propositions (incarnated as the products) offered by the producers (Vargo and Lusch, 2004). In the media domain, the core product—the content—can be altered, edited, and/or created by media users harnessing the enabling power of new media technology. Billions of new media users globally converge and diverge on every possible aspect of life, with traditional mass media outputs, constituting the entire media ecosystem. As discussed earlier, the broadcasting media age is platform driven—a few mass media platforms were able to draw most of the public attention, whereas the new media age is content driven—valuable content will flow across divergent media platforms, “it exists across platforms providing widely shared encounters that focus public attention on the most salient news and entertainment” (Webster, 2016, p. 163).

As a form of content, professional sport still attracts massive public attention (Vargo and Lusch, 2018). Along with the co-creation ability endowed by new media technology from sports fans and consumers, diversified content can be created to meet the highly individualized needs of distinct consumers. For example, for those fans who do not want to watch entire games that last 2–3 h long, they can search for game results, stats, and comments readily on various social media platforms (Singer, 2017). Some of those feeds are produced by official sources, others are created by regular fans and consumers themselves; combined, more content production equals more value to the old customers, and the more exposure, the more opportunity to draw the attention of new customers. Moreover, most of the user generated content generated *via* social media, is not profit-driven (Cova et al., 2015a,b). Therefore, professional sport can harness this content freely to augment the value of its product without worrying about competition to capture its value.

A content-driven paradigm also favors niche sports or entrepreneurs in the professional sport industry, brings other less prominent professional or even recreational sports to the forefront, and thus potentially alters the power dynamics of the professional sport industry. In a traditional mass media market, the most popular and powerful sport organizations and their products were prioritized to be broadcasted by dominant mass media providers. Whatever was broadcast by the mass media unequivocally attracted high levels of public attention. This mutual reinforcement mechanism resulted in a structure where only a few media companies and leagues/sports dominated. In this scenario, the importance of media income and low viewership and attention made niche sports struggle. In addition to receiving little media related income, niche sports also lacked media exposure, hence the public attention that further impeded their development (Billings, 2014). However, in the content-driven culture of the new media age, niche sports can at least create their own opportunities to promote their sports.

### The Strategic Paradox of Exploitation and Exploration

“Strategic paradoxes describe organization-level performing tensions that stem from the plurality of stakeholders and result in competing strategies and goals” (Smith, 2014, p. 1593). When facing radical technological change in the organizational environment, such as the emergence and proliferation of new media technology, growing complexity and uncertainty often exacerbate the pressures on organizations to reconcile simultaneously competing internal and external demands and develop the organization's strategic priorities (Greenwood et al., 2011; Besharov and smith, 2014; Smith, 2014). Managing strategic paradoxes is a formidable task because “pressure to minimize internal conflict and to address external legitimacy drive leaders to choose a single strategy” (Smith, 2014, p. 1594). Although strategy scholars have acknowledged that paradoxes are persistent and hard to resolve, some suggest that altering the “either/or” mindset to a paradoxical thinking of “both/and” (Smith and Tushman, 2005) can ease the tensions between conflicting requests. In this section, we introduce the strategic paradox of exploitation/ exploration as a major challenge that professional sports need to face in a complex cross media environment. Therefore, a balancing strategy which “involves defining a novel, creative synergy that addresses both oppositional elements together” (Smith, 2014, p. 1594) simultaneously can act as a means to manage the issues faced by professional sport in the digital age.

Balancing exploitation and exploration is a typical strategic paradox that can influence the decision making process of an organization (March, 1991; Benner and Tushman, 2003). The functional operation of an organization takes the cooperation and coordination of different units to work toward the same goal, and constant sensemaking and sensegiving (Gioia and Chittipeddi, 1991) between different units with divergent objectives and priorities increase operational costs and chances of conflict. Therefore, organizational routines have to be developed to keep the organization efficient and productive (Nelson and Winter, 1990). Often seen as a source of inertia and rigidity, strategy researchers have argued that organizational routines can facilitate change (Feldman and Pentland, 2003) as well and serve as the source of an organization's dynamic capability (Teece et al., 1997). Management techniques and procedures stemming from organizational routines can stimulate incremental changes and innovations that tend to answer to the demands of the existing market (March, 1991; Benner and Tushman, 2003). Incremental innovations focus managerial attention on exploiting an organization's existing resources and increase its ability to adapt to a stable environment “when technological environments are characterized by incremental refinements of an existing technological design” (Benner and Tushman, 2003, p. 249). Exploitation activities help the organization “find operational efficiencies in existing products for short-term sustainability” (Smith, 2014, p. 1593).

However, exploitation activities can hinder an organization's capability to engage in radical innovation, or exploration (March, 1991; Benner and Tushman, 2003), which “introduce novel innovations to achieve long-term sustainability (Smith, 2014, p. 1593). Exploration is important to organizations facing a turbulent environment with radical technological change and environmental uncertainty, because “the ability to develop new technological capabilities rapidly is especially critical in environments characterized by rapid innovation and change” (Benner and Tushman, 2003, p. 249). Therefore, in times of radical technological change in the organizational environment, the strategic paradox between exploitation and exploration becomes more prominent.

Professional sports institutionalized certain business routines to exploit opportunities in the age of broadcasting mass media (Gratton and Solberg, 2007; Milne, 2017), a period characterized by the dominance of the information economy. With limited media entities from which the audience could choose, and the uniformity those media provided (Webster, 2011), partnering with the broadcasting media could almost guarantee audience attention. As mentioned earlier, this ubiquitous pattern led to the creation of powerful national or even transnational professional sports entities (Webster, 2016). Initially, professional sports leagues and teams sold sports products and services directly to sports fans, gaining ticket revenue (Mason, 1999); second, sports leagues and teams sold the attention of fans to the media and sponsors alike, in exchange for broadcasting right fees and sponsorship revenue. Effective employment of this business model was sufficient to drive enormous revenue growth for the professional sport industry in the mass media age of the late twentieth Century (Zheng and Mason, 2018).

The proliferation of digital technology has stirred radical transitions which had an impact on the professional sport industry. This business model has been eroded by the transition from an information economy to an attention economy which facilitated online piracy and illegal streaming of sport games that challenged the exclusivity on which broadcast mass media relied to gain a high return on investment (Hutchins and Rowe, 2013a,b) and also changes to the viewing behaviors of profession sport consumers as described above. Facing the radical technological change of digitalization, many professional leagues and teams turned to incremental innovations in their existing business models, such as upgrading the transmission technology from an analog system to a high-quality digital paradigm, cooperating with online platforms to augment the sheer volume and diversity of the sport related content (Milne, 2017). They also promoted their brand, product, or those of the sponsors on social media platforms, trying to build and maintain long-term relationship with their fans and other customers. For example, the National Basketball Association (NBA) collaborates with online platforms to offer state-of-the-art services to basketball fans around the globe, beyond the basic live game television broadcast (Conway, 2014). In China, the NBA recently renewed its partnership with Tencent, one of the biggest Chinese digital media operators, and signed a new contract worth US $ 1.5 billion which runs through the 2024-25 season (NBA eyes Chinese growth in five-year, 2019).

Under the new deal, live NBA games, video on demand and short-video content will be available for fans through Tencent's digital and social media platforms including QQ.com, Tencent Sports, Tencent News, Tencent Video, QQ, Weixin/WeChat and Weshi. Tencent will develop innovative advertising products, and launch “mini programs” for mobile devices, including the NBA's fan loyalty program in China, “NBA Qiu Mi Quan.” (Frater, 2019, para. 2)^2^

However, these steps that the professional sport organizations took to adapt to the digitalization evolution are still characterized by non-radical innovations featuring a one-to-many mode customized for existing consumer sets (Benner and Tushman, 2003). Diversification and extension of the sport products does not change the fact that sport fans still assume a passive role as consumer with the professional sport organizations as the central active value producer, who resist embracing the co-creation power of the sport consumers *via* new media platforms with open arms (Hutchins and Rowe, 2013a,b). Future research should examine how the professional sport industry treats the agency created by social media users to generate additional value, while simultaneously circumventing risks stemming from such a dynamic and radically changing environment.

Moreover, Benner and Tushman (2003) suggested the establishment of an ambidextrous organization that can accommodate the need for operational efficiency and ferment innovation simultaneously. Specifically, an organization must both exploit existing technologies and efficiently execute incremental progression, while exploring new technology and effectively adapting to radical innovation; and these processes should be loosely coupled from each other so that functional efficiency will not hinder innovative effectiveness (Benner and Tushman, 2003). In the same vein, Zheng and Mason (2018) proposed a “combined multisided market and brand platform ecosystem” (p. 85) to deal with the paradox of exploitation and exploration from a macro-level perspective.

A multisided market can be exploited to adjust to incremental innovation under the one-to-many mode facilitated by digital technology, while a brand platform ecosystem should be a virtual-community-based, loosely coupled constellation of brand communities (Muniz and O'Guinn, 2001; Grant et al., 2011) subject to the autonomous oversight of various sports stakeholders, exploring radical changes sparked by the social media technology. In the brand platform ecosystem, professional sport experiments with radical innovation through trial and error, and shares co-created value with all its stakeholders. For example, NBA formed a strategic partnership with Kuaishou—a famous Chinese short video platform—on Oct 19, 2021 so that Kuaishou became “an Official NBA China Short Video Platform and the first Video Content Creation Community of NBA China” (Hubbard et al., 2018, para 1). In order to exploit and expand the brand value of NBA and Kuaishou,

Kuaishou and NBA will grant copyrighted content to outstanding creators and encourage users to make secondary creations. At the same time, this cooperation will give greater access to various rights and interests to high-quality content creators. Through the content creator backstage built by Kuaishou and the NBA, creators will be able to commercialize the creation of high-quality content. Kuaishou will provide promotion resources and commercialization opportunities for outstanding works, and jointly empower creators to monetize with platform commercial resources and NBA copyrighted content (Kuaishou Technology, 2021, para 5)

One promising future research avenue would be to empirically test the effectiveness of a brand platform ecosystem in facing and engaging in radical innovations, and its compatibility with a multisided market of professional sport.

### Organizational Social Approval Assets and New Media

Firm legitimacy, status, reputation and celebrity are crucial *social approval assets*— “intangible assets that derive their value from favorable stakeholder perceptions” (Hubbard et al., 2018)—that can bring the firm necessary resources to survive, develop and thrive in a highly competitive business environment by influencing key stakeholders' willingness to exchange resources with the firm (Deephouse, 2000; Lounsbury and Glynn, 2001; Rindova et al., 2006). Legitimacy emphasizes the importance of conforming behaviors for organizations in line with taken-for-granted norms and institutions derived from their institutional environment (Thornton et al., 2008). Organizational status reflects an organization's relative position in a networked environment “from accumulated acts of deference” (Sauder et al., 2012, p. 268), while firm reputation is considered the public discernment of an organization's outstanding capabilities based on its consistent prior performance (Deephouse, 2000; King and Whetten, 2008). Organizational celebrity is defined as a firm's ability to attract widespread public attention and to evoke positive affects at the same time (Rindova et al., 2006). Firm legitimacy, reputation, and status are analytical evaluations based on the rational judgement of stakeholders, while organizational celebrity is more emotion-driven (Pollock et al., 2019). However, new media—especially social media—renders constructs with dominant rational aspects, such as reputation, “becoming more emotional” and therefore more unpredictable (Pollock et al., 2019, p. 464). A single disruptive event can ferment on social media and rapidly spiral into a global-level event, in turn catching the attention of the mass media, continuing to escalate until eventually becoming a crisis that heavily impacts the focal firm's reputation in an unexpected manner (Etter et al., 2019).

For example, in 2019 a crisis related to the NBA's business relationship with China started when Daryl Morey, general manager of the Houston Rockets, tweeted his point of view on Hong Kong's state of political unrest. Despite the content's non-sport nature, and in spite of the fact that twitter has been banned from Chinese market, it instantaneously became viral on various Chinese social media platforms, triggering disaffection amongst NBA Chinese fans toward the Houston Rockets club. Many fans felt offended and asserted that the fact that Yao Ming had once played for the Rockets and the Rockets had been their favorite team since then made Morey's position even less acceptable. Emotional comments were left online, fans swore allegiance to their country, vowed to never watch Houston Rocket's games or even the NBA altogether, and requested Morey's dismissal. Ironically, after an initial response from the NBA, who expressed regret that Morey had deeply offended fans in China (Tensley, 2019), media in the US took it as a sign of weakness to submit to “China's money over human right” (Smith, 2019), whereas Chinese fans saw it as exuding arrogance and refusing to apologize. As the crisis continued to escalate Adam Silver, the commissioner of the NBA, had to reaffirm the NBA's position acknowledging Morey's right to freedom of speech to alleviate domestic tensions (Wade, 2019). However, in turn this statement further exacerbated Chinese fans' anger and drew derision from the Chinese mainstream media and even the Chinese government. Although this event has not been fully resolved, it has already caused some serious backlash such as the immediate suspension of partnerships from several major Chinese business sponsors, including the aforementioned agreement with Tencent. Tencent, “the NBA's exclusive digital partner in China,” elected to “suspend live streaming for the preseason games” (Tensley, 2019, para. 3). In addition, Chinese state television station (the CCTV) refused to broadcast NBA China preseason games as well (Tensley, 2019).

Importantly, the NBA's reputation has taken a heavy blow in China, which might potentially lead to more alienation of its biggest overseas market in the future, by an incident irrelevant to its prior business performance, or even outside the parameters of its business or sport performance entirely. This offers an intriguing research avenue awaiting future empirical examination. As the example above has shown, although social media enable regular media users to create user generated content and participate in the value co-creation process, which brings professional sport organization enormous opportunities; it also empowers fans and consumers with tools to engage in value co-destruction (Stieler et al., 2014). Value co-destruction is a nascent research topic in sport management study, thus requiring more in-depth empirical research to disclose the very nature of this phenomenon. In addition, more qualitative research—especially textual analysis—should be conducted to examine multiple social media platforms such as Facebook, Twitter or Instagram, in order to reveal how fans co-create or co-destruct value for the professional sport industry, or how the framing of discourse on social media platforms can influence intangible assets of the professional sport organization such as legitimacy, reputation, status or celebrity.

## More Future Research Avenues Opened up

Sport management research examining the influence of the digital transformation on the industry remains in its formative stages (Yoshida, 2017; Thompson et al., 2018). With more information competing for people's attention, and a corresponding finite attention span, sport fans might choose to pay more attention to certain highlights of the games that interest them, instead of watching an entire game thoroughly. The importance of the game itself might decline, as might the uncertainty of the game outcome, as the core product of professional sport in the mass media age (Mason, 1999). This transformation will likely reduce the importance of the strategies professional leagues adopted to keep competitive balance amongst teams (Fort, 1995). Future research could investigate empirically whether professional sport leagues start to emphasize (Lewis and Yoon, 2018) celebrity athletes at the expense of competitive balance, to what extent a player's online fame compensates for his/her on-court performance, and whether the longstanding uncertainty of outcome hypothesis (García and Rodríguez, 2002) begins to be undermined and replaced by a new hypothesis commensurate with a cross media regime, or remains intact even with the attention economy of the digital media age.

Further qualitative research is needed to theoretically ground how sport managers make decisions facing the inherent strategic paradox of exploitation and exploration (Smith, 2014). Other potential strategic paradoxes such as differentiation VS conformation, value creation VS value appropriation, internationalization VS localization, or the conflicting institutional logics of the market VS the community aggravated by a cross media complex could also be studied. For example, institutional logic research sheds light on how an organization manages co-existing competing institutional logics, and how frontline actors execute a great deal of agency to solve the paradox by cooperating with others with competing institutional logics to achieve a mutual goal on one hand, while maintaining strong independent identity on the other hand (McPherson and Sauder, 2000; Lounsbury, 2007; Reay and Hinings, 2009). In a professional sport context, empirical research should examine strategies and practices online marketing personnel employ to reconcile the competing institutional logics of the community derived from the formation of online virtual communities by fans and consumers, with the logics of the market imposed by the sport organization that they work for; and how the practices they undertake daily can result in the dominance of one particular logic, which will eventually cause field level institutional change (Smets et al., 2012).

## Conclusion

The professional sport industry achieved tremendous success in the traditional broadcast media age, established a multi-sided market and an effective business model for revenue growth. However, the emergence and proliferation of the new media technologies have drastically changed the media landscape, creating a much more complicated cross media environment that unites popularity and personalization, structure and agency (Webster, 2016). Such a changing environment creates transformations within the professional sport industry, and adapting to these transformations will lead to the evolution of the professional sport industry and its success in the digital media age. For established professional sport leagues and teams who already have a large fan base, co-creation can generate considerable value; for those entrepreneurial sports, digital media presents opportunities to break through barriers of a winner-take-all media market, so that their sport organizations can be exposed more to a highly variegated public attention. At the same time, radical technological change brings unprecedented conflict and uncertainty that can incur challenges and risks to even the most established professional sport organizations. Facing a much more complicated cross-media environment, the kind of dynamic capability (Teece et al., 1997) that professional sport organizations must foster remains worthy of sport management scholars' attention.

## Author Contributions

Both authors listed have made a substantial, direct, and intellectual contribution to the work and approved it for publication.

## Conflict of Interest

The authors declare that the research was conducted in the absence of any commercial or financial relationships that could be construed as a potential conflict of interest.

## Publisher's Note

All claims expressed in this article are solely those of the authors and do not necessarily represent those of their affiliated organizations, or those of the publisher, the editors and the reviewers. Any product that may be evaluated in this article, or claim that may be made by its manufacturer, is not guaranteed or endorsed by the publisher.

## Footnotes

^1^The movement of sports franchises from one city to another in order to obtain a more lucrative subsidy is a practice limited to North American markets, where leagues restrict the number of available franchises.

^2^This agreement has been undermined by recent events that have strained the relationship between the two parties.
